# The complete mitochondrial genome of *Hyotissasinensis* (Bivalvia, Ostreoidea) indicates the genetic diversity within Gryphaeidae

**DOI:** 10.3897/BDJ.11.e101333

**Published:** 2023-03-20

**Authors:** Fengping Li, Hongyue Liu, Xin Heng, Yu Zhang, Mingfu Fan, Shunshun Wang, Chunsheng Liu, Zhifeng Gu, Aimin Wang, Yi Yang

**Affiliations:** 1 State Key Laboratory of Marine Resource Utilization in South China Sea, Hainan University, Haikou, China State Key Laboratory of Marine Resource Utilization in South China Sea, Hainan University Haikou China; 2 College of Marine Science, Hainan University, Haikou, China College of Marine Science, Hainan University Haikou China; 3 Institute of Marine Science and Technology, Shandong University, Qingdao, China Institute of Marine Science and Technology, Shandong University Qingdao China; 4 Sanya Oceanographic Institution, Ocean University of China, Sanya, China Sanya Oceanographic Institution, Ocean University of China Sanya China; 5 Sanya Nanfan Research Institute, Hainan University, Sanya, China Sanya Nanfan Research Institute, Hainan University Sanya China

**Keywords:** Mitochondrial genome, gryphaeid oyster, gene order rearrangement, phylogeny

## Abstract

Different from the true oyster (family Ostreidae), the molecular diversity of the gryphaeid oyster (family Gryphaeidae) has never been sufficiently investigated. In the present study, the complete mitochondrial (mt) genome of *Hyotissasinensis* was sequenced and compared with those of other ostreoids. The total length of *H.sinensis* mtDNA is 30,385 bp, encoding 12 protein-coding-genes (PCGs), 26 transfer RNA (tRNA) genes and two ribosomal RNA (rRNA) genes. The nucleotide composition and codon usage preference of *H.sinensis* mtDNA is similar to that of *H.hyotis* within the same genus. On the other hand, the presence of three *trnM* and three *trnL* genes of *H.sinensis* was not detected neither in *H.hyotis* nor other ostroid species. Another unique character of *H.sinensis* mtDNA is that both *rrnS* and *rrnL* have a nearly identical duplication. The PCG order of *H.sinensis* is identical to *H.hyotis* and the two congener species also share an identical block of 12 tRNA genes. The tRNA rearrangements mostly happen in the region from *Cox1* to *Nad3*, the same area where the duplicated genes are located. The rearrangements within Gryphaeidae could be explained by a "repeat-random loss model". Phylogenetic analyses revealed Gryphaeidae formed by *H.sinensis* + *H.hyotis* as sister to Ostreidae, whereas the phylogenetic relationship within the latter group remains unresolved. The present study indicated the mitogenomic diversity within Gryphaeidae and could also provide important data for future better understanding the gene order rearrangements within superfamily Ostreoidea.

## Introduction

Oysters belong to superfamily Ostreoidea, which is comprised of Gryphaeidae and Ostreidae (Bouchet et al. 2010). Distributed worldwide, oysters are important fishery and aquaculture species (Guo et al. 2018). As the leading molluscan species by production, oysters have one of the longest cultured histories and remains cultured on all continents, except Antarctica (Botta et al. 2020). However, the oyster populations have declined throughout the world due to the influence of overfishing, habitat loss and degradation, disease and parasitic outbreaks (Wilberg et al. 2011). The protection and management of oyster resources depend on comprehensive information of genetic diversity at both species and population levels. With the development of molecular biology technologies, DNA sequences have been integrated into oyster identification to eliminate the influence from plasticity of shell shape and provide better understanding of oyster genetic diversity (Liu et al. 2011).

Previous studies have implied the effectiveness of mitochondrial DNA (mtDNA) as the molecular marker to reveal genetic diversity (Hebert et al. 2003). The mtDNA, especially the cytochrome c oxidase subunit 1 (COI) and the large ribosomal subunit (16S rDNA), has been applied in the species delimitation (Salvi et al. 2022), population genetics (Li et al. 2015) and phylogeographic analyses (Lazoski et al. 2011) of oyster resources. The complete mitochondrial genome which includes both the sequence and gene order information, has been widely used in oyster phylogenetic analyses (Salvi and Mariottini 2021). These previous studies revealed several mitogenomic characteristics within Ostreidae. Above all, the ostroid mitochondrial genome contains a split of the *rrnL* gene and a duplication of *trnM* (Danic-Tchaleu et al. 2011), compared with the typical metazoan mtDNA containing 13 protein coding genes (PCGs), two rRNA and 22 tRNA genes (Boore 1999). In the mtDNA of some Asian oysters, the duplications of the *rrnS* gene and *trnK* and *trnQ* genes have been disclosed (Wu et al. 2010). Compared with the tRNAs, the gene order of PCGs is more conserved amongst four genera (*Ostrea*, *Saccostrea*, *Magallana* and *Crassostrea*), despite some translocations and/or transversions happening between genera (Danic-Tchaleu et al. 2011). The gryphaeid oysters (family Gryphaedae) differ from the true oysters (family Ostreidae) in the morphology of larval shell and soft tissues (Bayne 2017). In addition, the vesicular microstructure of shell is uniquely found amongst Gryphaeidae species (Checa et al. 2020). Some gryphaeid species are also commercially important; however, their genetic diversity has seldom been well studied. Li et al. (2022) reported the mtDNA of *Hyotissahyotis* which represents the first complete mitochondrial genome within family Gryphaeidae. Different from the mitogenomic organisation of Ostreidae, neither the split of the *rrnL* nor the duplication of *trnM* was detected in that of *H.hyotis*. Furthermore, the PCG order of *H.hyotis* showed little shared gene blocks with ostroids, indicating that extensive rearrangements happened within superfamily Ostreoidea.

Despite the existence of one complete mitochondrial genome within Gryphaeidae, it is still necessary to include more data to conclude the mitogenomic features of this family. In the present study, the complete mitochondrial genome of *H.sinensis* was sequenced. Our aims are:

1. to characterise the mitogenomic features of *H.sinensis* and compare with *H.hyotis*;
2. to explore the gene order rearrangements within Gryphaeidae.

## Materials and methods

### Sample collection and DNA extraction

The specimen of *H.sinensis* was collected by scuba diving on the artificial fish reef in the marine ranching area of Wuzhizhou Island (18°18′55″N; 109°46′3″E). The adductor muscle of the specimen was deposited in 95% alcohol in the Laboratory of Economic Shellfish Genetic Breeding and Culture Technology (LESGBCT), Hainan University.

Whole genomic DNA was extracted from the adductor muscle of one individual using TIANamp Marine Animals DNA Kit (Tiangen, Beijing, China) in accordance with the manufacturer’s instructions. The genomic DNA was visualised on 1% agarose gel for quality inspection.

### DNA Sequencing and mitogenome assembly

Genomic DNA of *H.sinensis* was sent to Novogene (Beijing, China) for library construction and next-generation sequencing. The DNA library, with insert size of approximately 300 bp, was generated using NEB Next Ultra™ DNA Library Prep Kit for Illumina (NEB, USA) following the manufacturer’s instructions. It was then sequenced on the Illumina NovaSeq 6000 platform with 150 bp paired-end reads and 34,429,854 clean reads of each direction were finally generated. Clean data were imported in Geneious Prime 2021.0.1 for mitogenome assembly, with the strategy following Li et al. (2022). NOVOPlasty 4.2 (Dierckxsens et al. 2017) was also employed to avoid incorrect assembly.

Due to the existence of a duplicated region, which is more than 2,000 bp, this mitogenome is not able to be completely assembled only with the Illumina short reads. Therefore, a long PCR amplification was intended to fill the assembled gap using the 1F forward (5′-GGGGGTAAGATATTTTGTGCAGCGA-3′) and 1R reverse (5′-TCGACAGGTGGGCTAGACTTAACGC-3′) specific primers designed in the present study. The long PCR reactions contained 2.5 μl of 10× buffer (Mg^2+^ plus), 3 μl of dNTPs (2.5 mM), 0.5 μl of each primer (10 μM), 0.8 μl of template DNA (25–40 ng/μl), 0.2 μl of TaKaRa LA Taq DNA polymerase (5 U/μl) and DEPC (Diethypyrocarbonate) water up to 25 μl. Long PCR reactions were conducted by initial denaturation step at 94°C for 60 s, followed by 35 cycles of: 10 s at 98°C, 30 s at 57°C and 5 min at 68°C, then a final extension step at 68°C for 10 min. The PCR products were purified by ethanol precipitation and sequenced at Beijing Liuhe BGI (Beijing, China). The PCR primers were used as sequence primers.

### Mitogenomic annotation and sequence analysis

The mitogenome of *H.sinensis* was annotated using Geneious Prime. The PCGs were determined by ORF Finder (http://www.ncbi.nlm.nih.gov/orffinder) and MITOS Webserver (Bernt et al. 2013) with the invertebrate mitochondrial genetic code and their boundaries were modified by comparing them with those of congener species *H.hyotis* (GenBank Accession Number OP151093). The secondary structure of tRNA genes was predicted by MITOS and ARWEN (Laslett and Canbäck 2008), while the boundaries of rRNA genes were obtained using MITOS and modified according to those of other ostreoids.

The nucleotide composition of the whole complete mitogenome, PCGs, rRNA and tRNA genes was computed using MEGA X (Kumar et al. 2018). The base skew values for a given strand were determined as: AT skew = (A − T)/(A + T) and GC skew = (G − C)/(G + C), where A, T, G and C are the occurrences of the four nucleotides (Perna and Kocher 1995). Codon usage of PCGs was estimated using MEGA X. The mitochondrial genome map was generated using CGView (Grant and Stothard 2008).

### Phylogenetic analysis

A total of 21 ostreoid species was included for phylogenetic reconstruction (Table 1), with two pearl oysters *Pinctatamaxima* and *P.margaritifera* as outgroup following Plazzi and Passamonti (2010). The dataset concatenating the nucleotide sequences of the 12 PCGs (*Atp8* was not included) and two rRNA genes were constructed. The PCGs were aligned separately as codons using ClustalW integrated in MEGA X. The rRNA genes were aligned separately with MAFFT v.7 (Katoh and Standley 2013) and the ambiguously aligned positions were removed using Gblocks v.0.91b (Castresana 2000) with default parameters. The 14 separated alignments were finally concatenated into a single dataset using Geneious Prime and DAMBE5 (Xia 2018) was employed to generate different formats for further phylogenetic analyses. The best fit partition schemes and corresponding substitution models were identified using PartitionFinder 2 (Lanfear et al. 2017) under the Bayesian Information Criterion (BIC). The partitions tested in the present study referred to Li et al. (2022).

The Maximum Likelihood method (ML) and Bayesian Inference method (BI) were used for phylogenetic reconstruction. ML trees were constructed by IQtree 1.6.12 (Nguyen et al. 2015), which allows different partitions to have different evolutionary rates (-spp option) and with 10,000 ultrafast bootstrap replicates (-bb option). BI trees were constructed using MrBayes v.3.2.6 (Ronquist et al. 2012), running four simultaneous Monte Carlo Markov Chains (MCMC) for 10,000,000 generations, sampling every 1000 generations and discarding the first 25% generations as burn-in. Two independent runs were performed to increase the chance of adequate mixing of the Markov chains and to increase the chance of detecting failure to converge, as determined using Tracer v.1.6. The effective sample size (ESS) of all parameters was above 200. The generated phylogenetic trees were visualised in FigTree v.1.4.2.

## Results and discussion

### Species identification and mitogenome assembly

**Figure 1.**
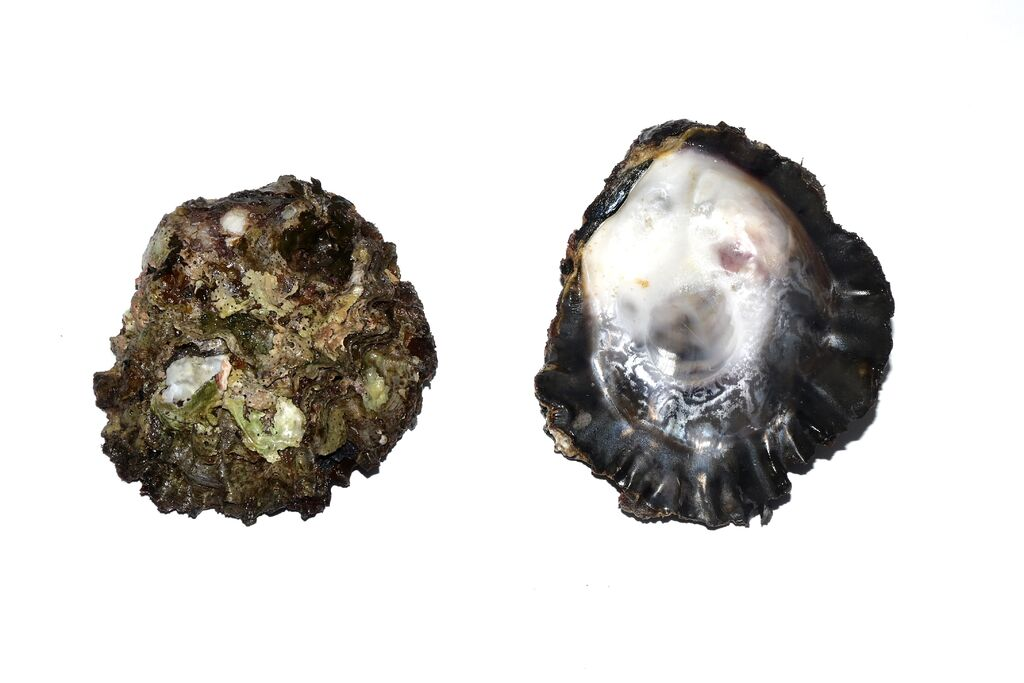
The image of *Hyotissasinensis*.

Misidentifications are quite frequent in oyster mitogenomics. This is the case for the example of the recently-published mitogenome of *Alectryonellaplicatula* (with GenBank Accession Number MW143047) that, in fact, was found to be a misidentified *Magallanagigas* as reported by Salvi et al. (2021). The identification of *H.sinensis* was conducted, based on both morphological and molecular evidence. The specimen in the present study possesses an oval shell with a length of about 14 cm (Fig. 1). The shell surface irregularly folds with radial ribs on both valves, which are weaker than those of *H.hyotis*. The margin of interior shell is dark purple, while the central part is white. The adductor muscle scar is large and located at the posterior side of the centre of the shell. Molecular identification was following Salvi et al. (2021), based on the *rrnL* fragment, which shows identity values from 99.19% to 99.79% to the previously published sequences on GenBank (KC847135 and MT332230).

This mitogenome was firstly assembled. based on the next-generation data using two different types of software, resulting in almost identical results. However, two repetitive sequences that corresponded to the partial *rrnL* and *rrnS* genes were discovered on both sides of the draft mitogenome, indicating the incomplete assembly derived from short Illunima sequencing reads. The long PCR amplification which generated a product with 3,068 bp in length finally covered the assembly gap and completed the duplicated *rrnL* and *rrnS* genes. No additional gene was discovered within this Sanger-sequencing fragment.

### Mitochondrial genome composition

**Figure 2.**
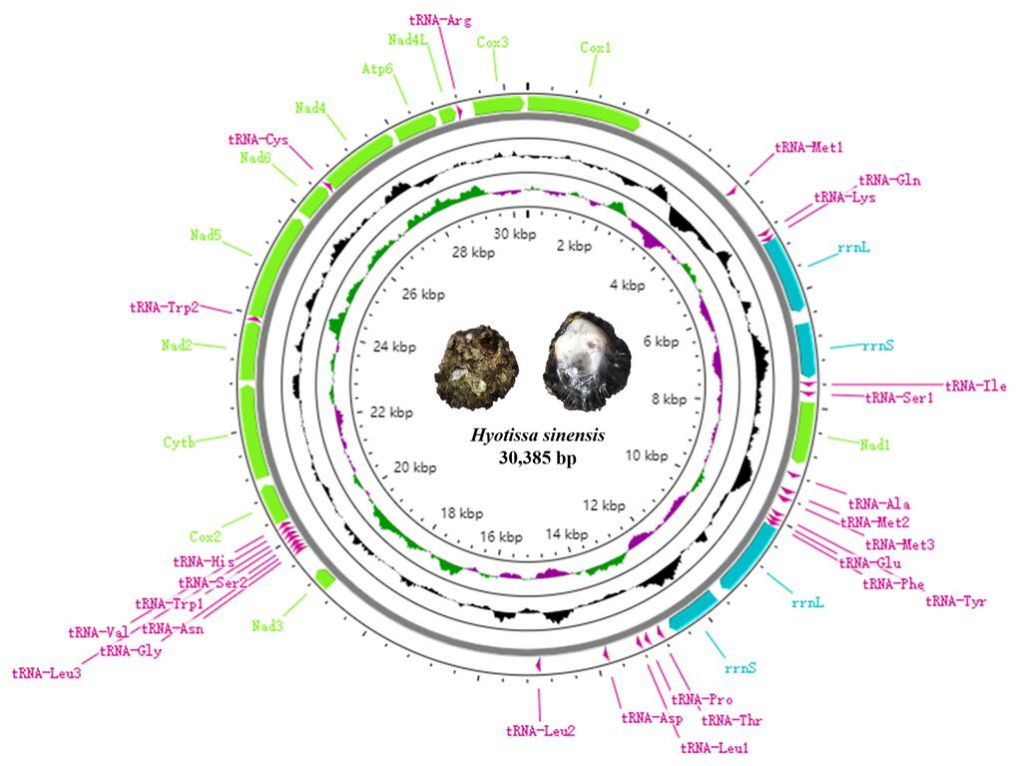
Mitochondrial genome map of *Hyotissasinensis*.

**Figure 3.**
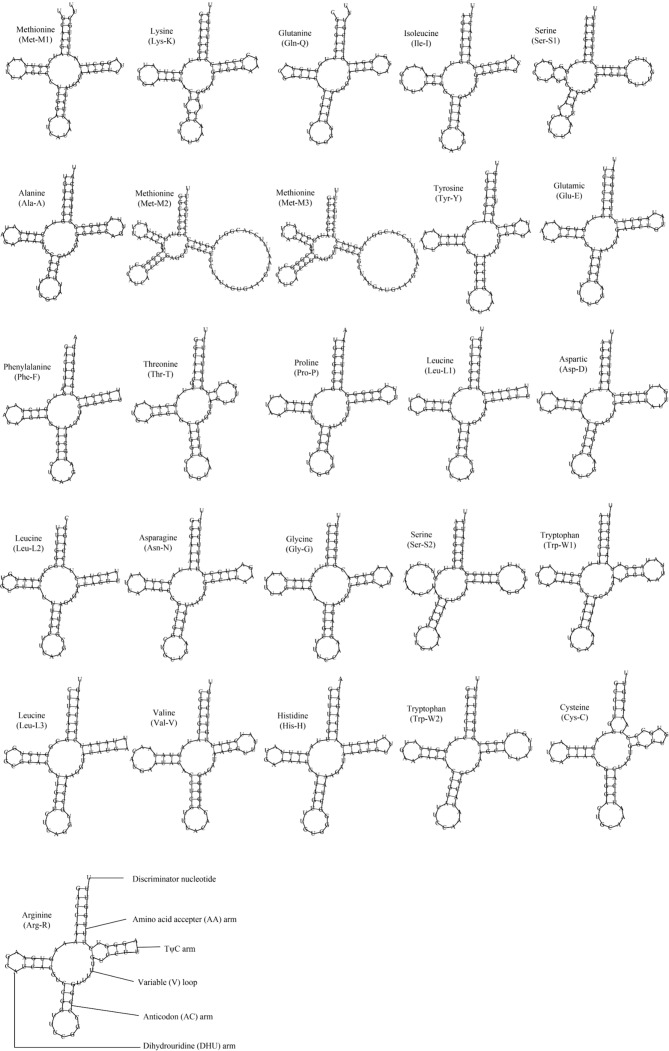
Inferred secondary structures of 26 transfer RNAs from *Hyotissasinensis*.

The total length of *H.sinensis* mtDNA is 30,385 bp, encoding 40 genes including 12 PCGs, 26 tRNA genes and two rRNA genes (Table 2). The size of *H.sinensis* mitogenome is obviously longer than the other species from superfamily Ostreoidea (Wu et al. 2010, Li et al. 2022). All mitochondrial genes of *H.sinensis* are encoded on the same strand (Fig. 2), as previously indicated in other marine bivalves (Ghiselli et al. 2021). Different from the typical metazoan mtDNA, the *Atp8* gene is not detected in *H.sinensis*. Although *Atp8* was found in *H.hyotis* (Li et al. 2022), the identification of this short sequence is laborious due to its high substitution rate that led to the low homology even to its congener species. Although the absence of the *Atp8* gene in family Ostreidae was reported by Ren et al. (2010), subsequent studies discovered this ATP gene in oyster mitogenome where it was thought to be absent (Wu et al. 2012). Amongst the 26 tRNAs of *H.sinensis*, three *trnM* were discovered (Fig. 3). In addition, *H.sinensis* consists of one extra copy of *trnL*-UUR and one of *trnW*. Another unique character is that both *rrnS* and *rrnL* have a nearly identical duplication (Fig. 2). The largest non-coding region located between *trnL* and *Nad3* is 3,901 bp in length (Table 2).

The overall AT content of the *H.sinensis* mtDNA is 57.2%, similar to that of *H.hyotis* (59.2%; Li et al. (2022)). The AT skew and GC skew are -0.15 and 0.27, respectively (Table 3), indicating that the nucleotide composition is skewed from A in favour of T and from C to G. The negative AT skew and positive GC skew have also been reported in other ostreoid mitogenomes (Wu et al. 2010).

### PCGs, tRNA and rRNA genes

The AT content of the concatenated PCGs is 57.0% (Table 3). Amongst the individual PCGs, the AT content values range from 54.7% (*Nad4L*) to 60.3% (*Atp6*). The AT and GC skews of PCGs also show the same tendency of asymmetry as the mitogenome.

**Figure 4.**
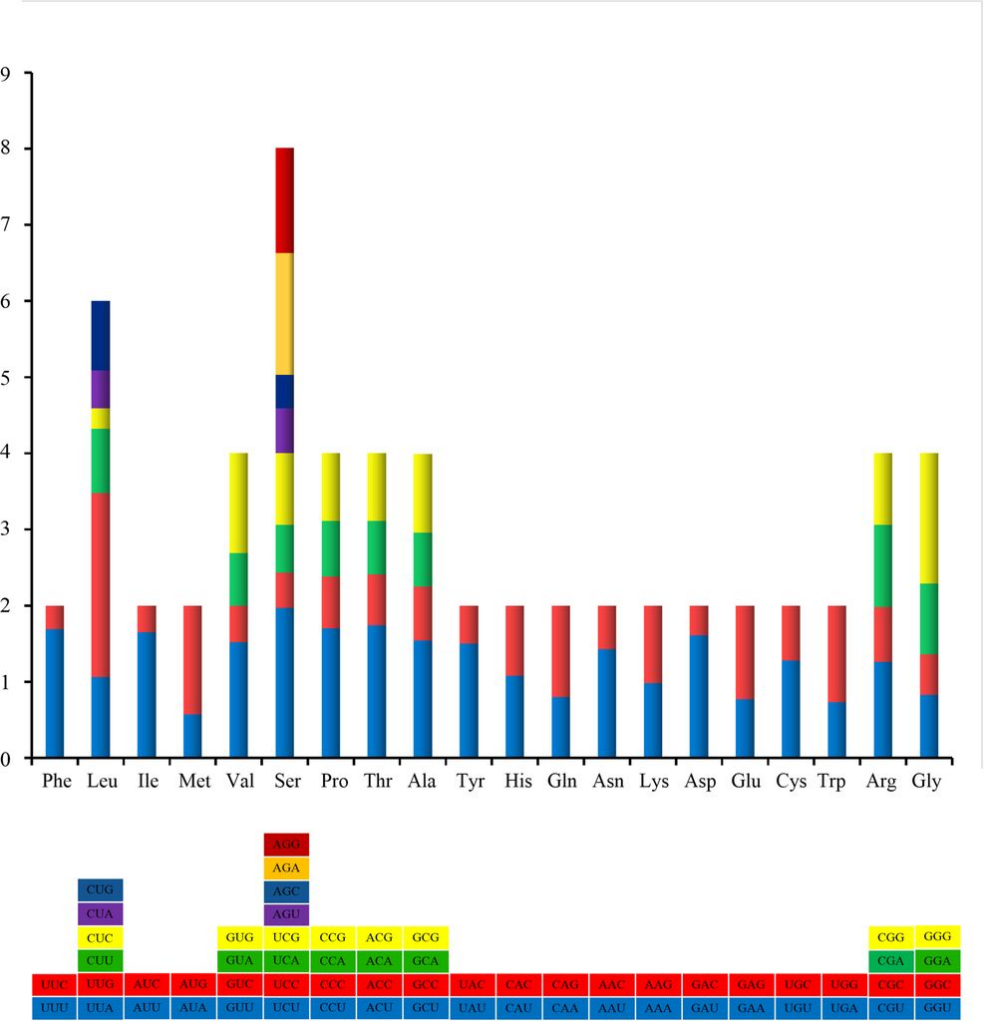
Relative synonymous codon usage (RSCU) of mitochondrial genome for *Hyotissasinensis*.

The PCG start/stop codon usage preference of *H.sinensis* is different from that of *H.hyotis*. Amongst the 12 PCGs, seven genes start with the conventional initiation codons ATG (*Cox1*, *Nad1*, *Cox2*, *Nad6*, *Nad4* and *Atp6*) and ATA (*Cytb*). The alternative start codons ATT (*Nad2* and *Nad3*), TTG (*Nad4L* and *Nad5*) and TTT (*Cox3*) are detected in the remaining five genes. All PCGs employ the conventional stop codons TAA (*Cox1*, *Cox3* and *Nad2*) and TAG, except for *Nad3* which use the truncated stop codon T. The incomplete stop codons (TA and T) could be presumably modified to TAA through post-transcriptional polyadenylation (Ojala et al. 1981). The relative synonymous codon usage (RSCU) values of *H.sinensis* are shown in Table 4. Amongst all the amino acids, the frequency of leucine is the highest, as suggested in *H.hyotis* as well as in other invertebrate groups (Sun et al. 2020, Yang et al. 2020). Significant synonymous codon usage bias is also observed in the PCGs of *H.sinensis*, similar to that of *H.hyotis* (Fig. 4). Most of the preferred codons (e.g. TTT and TTG) are composed of T and G, which could explain the negative AT skew and positive GC skew of the PCGs to some extent.

**Figure 5.**
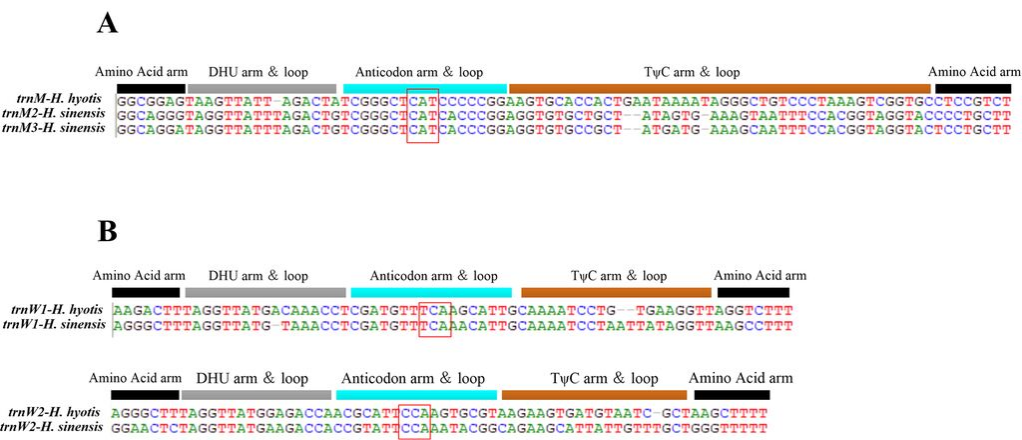
Alignment of *trnM* sequences (A) and *trnW* (B) in mitochondrial genomes of *Hyotissasinensis* and *H.hyotis*. tRNA secondary structure is displayed above the alignment and the position of the anticodon is highlighted within the rectangular frame.

The AT content of the concatenated tRNAs is 56.3%, while the AT skew and GC skew are -0.14 and 0.19, respectively (Table 3). The length of tRNA genes ranges from 63 to 89 bp (Table 2). All the 26 tRNA genes could be folded into typical clover-leaf secondary structures, except for *trnS*-UCN and *trnS*-AGN which lack the dihydrouracil (DHU) arm, but are simplified down to a loop (Fig. 3). The missing DHU arm in the secondary structure of *trnS*-AGN is quite common in metazoan mitogenomes (Wolstenholme 1992). However, lack of the DHU arm in *trnS*-UCN is not a common feature observed in invertebrate mitogenomes, though it has been found in some arthropod taxa (Wang et al. 2016). The typical metazoan mtDNA possesses a total of 22 tRNA genes, including two copies of *trnL* and two of *trnS*. However, the bivalve mtDNA usually shows deviations especially in the number of tRNAs. A typical example is the existence of one extra *trnM* in most bivalve mitogenomes (Lee et al. 2019, Wang et al. 2021). The presence of three *trnM* in *H.sinensis* has never been reported in Ostreoidea before. All three *trnM* genes recognise codon AUG, but *trnM*2 and *trnM*3 share almost identical sequences, indicating the evidence of the tRNA duplication event which happens quite commonly in molluscan mitogenomes (Ghiselli et al. 2021). Sequence comparison suggests that *trnM*2 and *trnM*3 in *H.sinensis* are homologous to the single *trnM* in *H.hyotis* (Fig. 5). Amongst the three *trnL*, two copies that recognise the codon UUA indicate another case of tRNA duplication (Fig. 3). The two *trnW* genes in *H.sinensis* could also be traced in *H.hyotis* (Fig. 5). The appearance of two *trnW* genes that occur only in Gryphaeidae should be considered as an occasional event within Ostreoidea (Wu et al. 2012).

The *H.sinensis* contains two almost identical copies of *rrnL* and two of *rrnS*, which were not detected in *H.hyotis* (Li et al. 2022). The duplication of *rrnS* is considered as a common feature of the Asian genus *Magallana*. Similarly, it is assumed that the duplication of *rrnL* and *rrnS* in *H.sinensis* is a derived character, but still needs to be further determined by the inclusion of more data within Gryphaeidae. The two copies of *rrnS* are 944 bp in length, while the two *rrnL* copies are 1,329 bp and 1,366 bp, respectively. The AT content, AT skew and GC skew values of rRNA genes are shown in Table 3.

### Phylogenetic analyses

**Figure 6.**
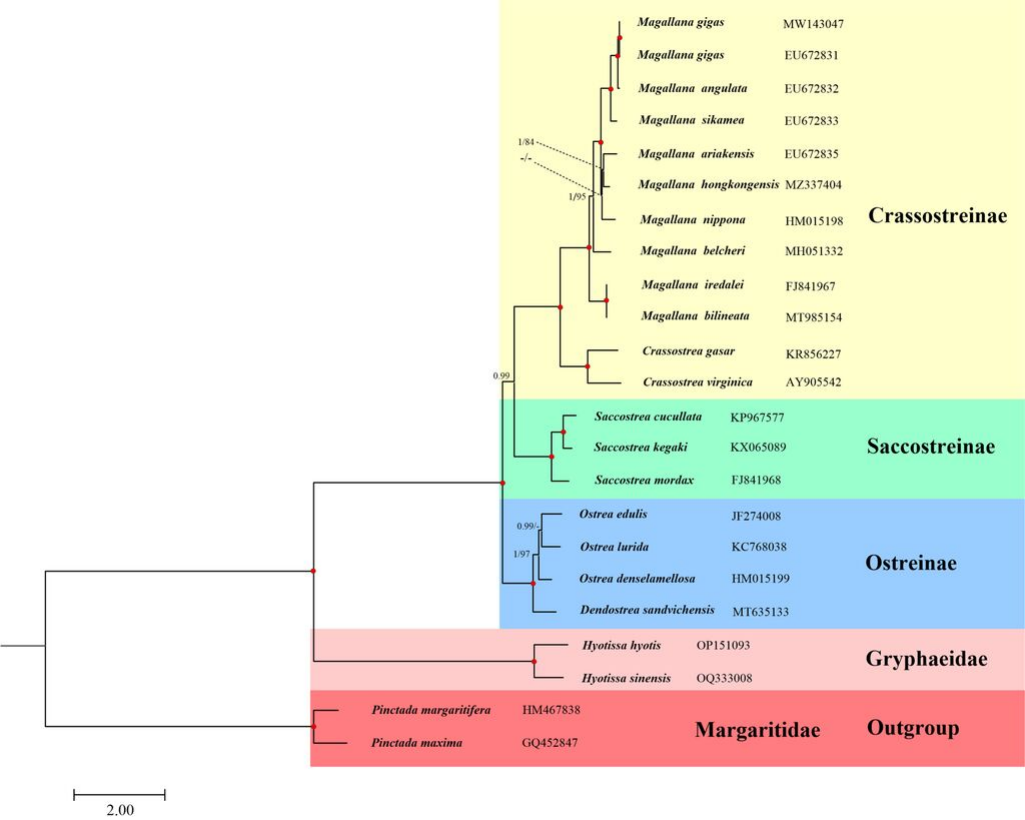
Phylogenetic relationships of Ostreoidea, based on the concatenated nucleotide sequences of 12 mitochondrial protein-coding genes and two ribosomal RNA genes. The reconstructed Bayesian Inference (BI) phylogram is shown. The first number at each node is Bayesian posterior probability (PP) and the second number is the bootstrap proportion (BP) of Maximum Likelihood (ML) analyses. The nodal with maximum statistical supports (PP = 1; BP = 100) is marked with a solid red circle. BP values under 80 and PP values under 0.90 are marked as a dash line.

According to the BIC, the best partition scheme is the one combining genes by subunits, but analysing each codon position separately (Suppl. material 1). ML (−lnL = 154,942.703) and BI (−lnL = 150,197.79 for run 1; −lnL = 150,198.99 for run 2) analyses arrived at almost identical topologies (Fig. 6). Within Ostreoidea, Gryphaeidae formed by *H.hyotis* and *H.sinensis* was recovered as sister to Ostreidae. Different from the extinct gryphaeids which have been widely researched (Dietl et al. 2000, Hautmann et al. 2017, Kosenko 2019), only a few studies focused on the living gryphaeids. Based on several short gene fragments, Li et al. (2021) reconstructed the phylogenetic relationships of Ostreoidea, within which the monophyletic *Hyotissa* (including both *H.hyotis* and *H.sinensis*) was sister to *Pycnodonte* + *Neopycnodonte* despite the poor support values at some points. However, future studies with the inclusion of broader mitogenomic data are still needed to solve the phylogenetic relationships of Gryphaeidae.

The phylogenetic relationships within Ostreidae generated from ML and BI methods arrived at different topologies (Fig. 6). The BI tree in the present study is consistent with Li et al. (2022), in which the rRNA genes were not included, while the ML tree here suggests Crassostreinae as sister to (Ostreinae + Saccostreinae), which is supported by previous phylogenies (Danic-Tchaleu et al. 2011, Salvi et al. 2014). This controversy has been discussed by Li et al. (2022). In addition to the inclusion of rRNA genes for phylogenetic reconstruction, this study also included the mitogenomic data of genus *Dendostrea* compared with Li et al. (2022). Firstly, *Dendostreasandvichensis* and its sister group *Ostrea* constitute subfamily Ostreinae, which is in accordance with the current classification (MolluscaBase 2023). Morphologically, *Dendostrea* species could be distinguished from its radiating ribs on the surface of the right valve (Hu et al. 2019).

**Figure 7.**
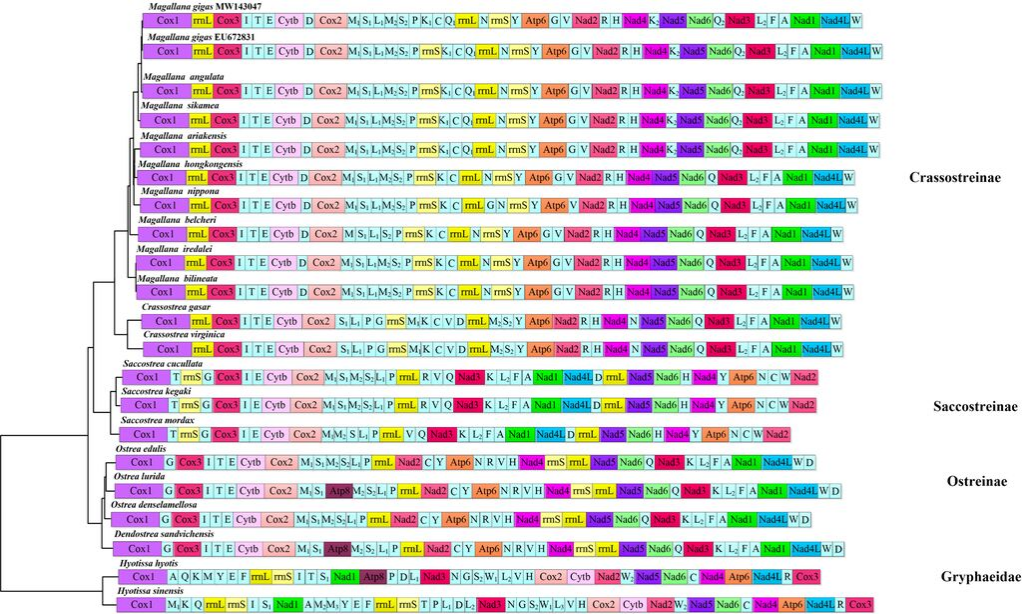
Linearised PCG order of Ostreoidea, based on the phylogenetic tree.

Within Crassostreinae, two separated clades corresponding to the Asia-Pacific and Atlantic Regions are clearly presented (Figs 6, 7). Recently, the Pacific cupped oysters which were previously included in *Crassostrea* along with the Atlantic cupped oysters, were re-assigned to genus *Magallana* (Salvi and Mariottini 2017). Subsequent studies have demonstrated that *Magallana* was well-founded, based on a scientific basis and its validity has been thoroughly discussed (Willan 2021, Salvi and Mariottini 2021, Spencer et al. 2022). The present phylogeny also provides support for this classification.

### Gene rearrangement

Within Ostreidae, the gene rearrangement events are most common in tRNA genes (Ren et al. 2010). Although some shared PCG blocks could be detected amongst the four ostreid genera, *Magallana*, *Crassostrea*, *Ostrea* and *Saccostrea*, it is still not possible to assume a pleisomorphic gene order in Ostreidae, based on available data as discussed in Salvi and Mariottini (2021). Within Ostreoidea, one shared gene block (*Nad5*-*Nad6*-*Nad4*-*Atp6*), plus one inverted gene block (*Nad1*-*Nad3*-*Cox2*-*Cytb*) were detected between *H.hyotis* (Gryphaeidae) and *Saccostrea* (Ostreidae). The newly-sequenced mitogenome of *H.sinensis* further confirms this feature of Gryphaeidae. Above all, the PCG order of *H.sinensis* (excluding the *ATP8* gene since it is missing in *H.sinensis*) is identical to *H.hyotis* (Fig. 7), in agreement with the pattern that PCG order is conserved within the genus as mentioned in Ostreidae. Furthermore, *H.sinensis* also shares an identical block of 12 tRNAs with *H.hyotis* (Fig. 7). The tRNA rearrangements mostly happen in the region from *Cox1* to *Nad3*, the same area where the duplicated genes are located. As a result, the rearrangements within Gryphaeidae could be explained by a "repeat-random loss model" (San Mauro et al. 2005). To understand how the PCG orders evolved within superfamily Ostreoidea, more mitogenomes belonging to Gryphaeidae (including genera *Pycnodonte* and *Neopycnodonte*), as well as a robust phylogenetic framework, are still needed.

## Supplementary Material

33046E34-4758-5891-ADD6-64764E9C81BE 10.3897/BDJ.11.e101333.suppl1 Supplementary material 1 Best fit partitions and substitution models
Data type Table File: oo_801937.docx https://binary.pensoft.net/file/801937 Fengping Li, Yi Yang

## Figures and Tables

**Table 1.** List of mitochondrial genomes used in this study.

**New mt genomes**							
**Family**		**Species**	**Length (bp)**		**Sampling time**		**Accession No.**
Gryphaeidae		*Hyotissasinensis*	30,385		June 2022		OQ333008
**GenBank mt genome**							
**Family**	**Species**			**Length (bp)**		**Accession No.**	
Gryphaeidae	*Hyotissahyotis*			22,185		OP151093	
Ostreidae	*Dendostreasandvichensis*			16,338		MT635133	
Ostreidae	*Magallanagigas*			18,225		MW143047	
Ostreidae	*Magallanagigas*			18,225		EU672831	
Ostreidae	*Magallanahongkongensis*			18,617		MZ337404	
Ostreidae	*Magallanabilineata*			22,420		MT985154	
Ostreidae	*Magallanabelcheri*			21,020		MH051332	
Ostreidae	*Magallananippona*			20,030		HM015198	
Ostreidae	*Magallanairedalei*			22,446		FJ841967	
Ostreidae	*Magallanaariakensis*			18,414		EU672835	
Ostreidae	*Magallanasikamea*			18,243		EU672833	
Ostreidae	*Magallanaangulata*			18,225		EU672832	
Ostreidae	*Crassostreagasar*			17,685		KR856227	
Ostreidae	*Crassostreavirginica*			17,244		AY905542	
Ostreidae	*Ostreadenselamellosa*			16,277		HM015199	
Ostreidae	*Ostreaedulis*			16,320		JF274008	
Ostreidae	*Ostrealurida*			16,344		KC768038	
Ostreidae	*Saccostreamordax*			16,532		FJ841968	
Ostreidae	*Saccostreacucullata*			16,396		KP967577	
Ostreidae	*Saccostreakegaki*			16,260		KX065089	
Margaritidae	*Pinctadamargaritifera*			15,680		HM467838	
Margaritidae	*Pinctadamaxima*			16,994		GQ452847	

**Table 2.** Gene annotations of the complete mt genome of *Hyotissasinensis*.

**Gene**	**Strand**	**Location**	**Size (bp)**	**Start Codon**	**Stop codon**	**Intergenic nucleotides**
*Cox1*	H	1-1986	1986	ATG	TAA	1936
tRNA-*Met1*	H	3923-3989	67			842
tRNA-*Lys*	H	4832-4898	67			81
tRNA-*Gln*	H	4944-5006	63			0
*rrnL*	H	5007-6335	1329			212
*rrnS*	H	6548-7491	944			74
tRNA-*Ile*	H	7566-7632	67			97
tRNA-*Ser1*	H	7730-7799	70			105
*Nad 1*	H	7905-8978	1074	ATG	TAG	185
tRNA-*Ala*	H	9164-9230	67			220
tRNA-*Met2*	H	9451-9539	89			54
tRNA-*Met3*	H	9594-9682	89			203
tRNA-*Tyr*	H	9886-9948	63			37
tRNA-*Glu*	H	9986-10051	66			9
tRNA-*Phe*	H	10061-10125	65			16
*rrnL*	H	10142-11507	1366			192
*rrnS*	H	11700-12643	944			164
tRNA-*Thr*	H	12808-12870	63			179
tRNA-*Pro*	H	13050-13115	66			91
tRNA-*Leu1*	H	13207-13269	63			537
tRNA-*Asp*	H	13807-13874	68			1112
tRNA-*Leu2*	H	14987-15049	63			3901
*Nad3*	H	18951-19290	340	ATT	T	444
tRNA-*Asn*	H	19735-19801	67			2
tRNA-*Gly*	H	19804-19869	66			7
tRNA-*Ser2*	H	19877-19946	70			25
tRNA-*Trp1*	H	19972-20039	68			13
tRNA-*Leu3*	H	20053-20115	63			29
tRNA-*Val*	H	20145-20211	67			30
tRNA-*His*	H	20242-20305	64			27
*Cox2*	H	20333-21028	696	ATG	TAG	125
*Cytb*	H	21154-22785	1632	ATA	TAG	78
*Nad2*	H	22864-23883	1020	ATT	TAA	14
tRNA-*Trp2*	H	23898-23965	68			6
*Nad5*	H	23975-25828	1857	TTG	TAG	96
*Nad6*	H	25925-26527	603	ATG	TAG	30
tRNA-*Cys*	H	26558-26620	63			1
*Nad4*	H	26622-27965	1344	ATG	TAG	74
*Atp6*	H	28040-28786	747	ATG	TAG	
*Nad4L*	H	28855-29145	291	TTG	TAG	35
tRNA-*Arg*	H	29181-29243	63			209
*Cox3*	H	29453-30331	879	TTT	TAA	54

**Table 3.** List of AT content, AT skew and GC skew of *Hyotissasinensis* mtDNA.

Feature	(A+T)%	AT skew	GC skew
Whole genome	57.2	-0.15	0.27
PCGs	57.0	-0.23	0.29
PCGs1	55.7	-0.24	0.40
PCGs2	56.4	-0.13	0.28
PCGs3	58.7	-0.31	0.19
*Atp6*	60.3	-0.26	0.39
*Cox1*	58.4	-0.16	0.24
*Cox2*	57.8	-0.25	0.29
*Cox3*	56.2	-0.30	0.20
*Cytb*	57.1	-0.16	0.23
*Nad1*	55.8	-0.18	0.21
*Nad2*	57.5	-0.29	0.26
*Nad3*	55.8	-0.35	0.44
*Nad4*	55.5	-0.30	0.34
*Nad4L*	54.7	-0.17	0.45
*Nad5*	56.3	-0.25	0.37
*Nad6*	56.2	-0.15	0.39
tRNAs	56.3	-0.14	0.19
*rrnL1*	57.6	-0.03	0.20
*rrnS1*	52.8	0.09	0.13
*rrnL2*	58.4	-0.02	0.20
*rrnS2*	52.6	0.10	0.13

**Table 4.** Codon and relative synonymous codon usage (RSCU) of 12 protein-coding genes (PCGs) in the mtDNA of *Hyotissasinensis*.

Amino Acid	Codon	Count (RSCU)	Amino Acid	Codon	Count (RSCU)
Phe	UUU	254.0(1.69)	Ala	GCU	101.0(1.54)
	UUC	47.0(0.31)		GCC	47.0(0.71)
Leu	UUA	83.0(1.06)		GCA	47.0(0.71)
	UUG	189.0(2.42)		GCG	68.0(1.03)
	CUU	66.0(0.84)	Gly	GGU	73.0(0.83)
	CUC	21.0(0.27)		GGC	47.0(0.53)
	CUA	39.0(0.50)		GGA	82.0(0.93)
	CUG	71.0(0.91)		GGG	151.0(1.71)
Ile	AUU	191.0(1.65)	Arg	CGU	35.0(1.26)
	AUC	40.0(0.35)		CGC	20.0(0.72)
Met	AUA	63.0(0.57)		CGA	30.0(1.08)
	AUG	159.0(1.43)		CGG	26.0(0.94)
Val	GUU	151.0(1.52)	Tyr	UAU	136.0(1.50)
	GUC	48.0(0.48)		UAC	45.0(0.50)
	GUA	69.0(0.69)	His	CAU	47.0(1.08)
	GUG	130.0(1.31)		CAC	40.0(0.92)
Ser	UCU	90.0(1.97)	Gln	CAA	22.0(0.80)
	UCC	21.0(0.46)		CAG	33.0(1.20)
	UCA	29.0(0.63)	Asn	AAU	77.0(1.43)
	UCG	43.0(0.94)		AAC	31.0(0.57)
	AGU	27.0(0.59)	Lys	AAA	86.0(0.98)
	AGC	20.0(0.44)		AAG	89.0(1.02)
	AGA	73.0(1.60)	Asp	GAU	75.0(1.61)
	AGG	63.0(1.38)		GAC	18.0(0.39)
Pro	CCU	65.0(1.70)	Glu	GAA	57.0(0.77)
	CCC	26.0(0.68)		GAG	92.0(1.23)
	CCA	28.0(0.73)	Cys	UGU	64.0(1.28)
	CCG	34.0(0.89)		UGC	36.0(0.72)
Thr	ACU	70.0(1.74)	Trp	UGA	62.0(0.73)
	ACC	27.0(0.67)		UGG	107.0(1.27)
	ACA	28.0(0.70)	*	UAA	3.0(0.55)
	ACG	36.0(0.89)		UAG	8.0(1.45)
