# Supplementary material for: The complete mitochondrial genome of Hyotissasinensis (Bivalvia, Ostreoidea) indicates the genetic diversity within Gryphaeidae
Source: Biodivers Data J. 2023 Mar 20;11:e101333. doi: 10.3897/BDJ.11.e101333 (PMC10848854; doi:10.3897/BDJ.11.e101333)
Supplement: Supplementary material 1 — Best fit partitions and substitution models [file bdj-11-e101333-s001.docx]

Table S1. Best fit partitions and substitution models.

|  | Set Partition (Conducted by PartitionFinder 2) | Best Model for BI (Selected by PartitionFinder 2) | Best Model for ML (Selected by ModelFinder) |
| --- | --- | --- | --- |
| Best Partition CDS genes | *Atp6* 1th | TRN+G | TN+F+G4: |
| (BIC= 286439.89) | *Atp6* 2th | TVM+G | TPM3+F+G4 |
|  | *Atp6* 3th | TRN+G | TPM2+F+G4 |
|  | *Cox1-2-3* 1th | TRN+G | TIM2+F+I+G4 |
|  | *Cox1-2-3* 2th | GTR+G | TVM+F+R3 |
|  | *Cox1-2-3* 3th | TRN+I+G | HKY+F+R6 |
|  | *Cytb* 1th | TRN+G | TN+F+G4 |
|  | *Cytb* 2th | TVM+G | TVM+F+G4 |
|  | *Cytb* 3th | TRN+I+G | HKY+F+R6 |
|  | *Nad1-2-3-4-4L-5-6* 1th | GTR+I+G | TIM2+F+I+G4 |
|  | *Nad1-2-3-4-4L-5-6* 2th | TVM+G | TVM+F+G4 |
|  | *Nad1-2-3-4-4L-5-6* 3th | TRN+I+G | TN+F+R6 |
| rRNA genes (BIC=16448.64) | *rrnS-rrnL* | TVM+G | GTR+F+R3 |
